# NMF-Based Approach for Missing Values Imputation of Mass Spectrometry Metabolomics Data

**DOI:** 10.3390/molecules26195787

**Published:** 2021-09-24

**Authors:** Jingjing Xu, Yuanshan Wang, Xiangnan Xu, Kian-Kai Cheng, Daniel Raftery, Jiyang Dong

**Affiliations:** 1 Department of Electronic Science, Xiamen University, Xiamen 361005, China; jingjing@xmu.edu.cn (J.X.); wangyuanshan@sfmail.sf-express.com (Y.W.); 2 School of Mathematics and Statistics, The University of Sydney, Sydney, NSW 2006, Australia; xiangnan.xu@sydney.edu.au; 3 Innovation Centre in Agritechnology, Universiti Teknologi Malaysia, Johor, Muar 84600, Malaysia; chengkiankai@cheme.utm.my; 4 Northwest Metabolomics Research Center, Department of Anesthesiology and Pain Medicine, University of Washington, Seattle, WA 98109, USA; draftery@uw.edu; 5 National Institute for Data Science in Health and Medicine, Xiamen University, Xiamen 361005, China

**Keywords:** non-negative matrix factorization, missing values imputation, mass spectrometry, metabolomics data, missing pattern, outliers

## Abstract

In mass spectrometry (MS)-based metabolomics, missing values (NAs) may be due to different causes, including sample heterogeneity, ion suppression, spectral overlap, inappropriate data processing, and instrumental errors. Although a number of methodologies have been applied to handle NAs, NA imputation remains a challenging problem. Here, we propose a non-negative matrix factorization (NMF)-based method for NA imputation in MS-based metabolomics data, which makes use of both global and local information of the data. The proposed method was compared with three commonly used methods: k-nearest neighbors (kNN), random forest (RF), and outlier-robust (ORI) missing values imputation. These methods were evaluated from the perspectives of accuracy of imputation, retrieval of data structures, and rank of imputation superiority. The experimental results showed that the NMF-based method is well-adapted to various cases of data missingness and the presence of outliers in MS-based metabolic profiles. It outperformed kNN and ORI and showed results comparable with the RF method. Furthermore, the NMF method is more robust and less susceptible to outliers as compared with the RF method. The proposed NMF-based scheme may serve as an alternative NA imputation method which may facilitate biological interpretations of metabolomics data.

## 1. Introduction

Mass spectrometry (MS) is a widely-used analytical technique for the profiling and analysis of small molecule metabolites, due to its high sensitivity, high throughput, and high resolution advantages [1]. However, data collected from an MS instrument may contain 10–20% missing values (NAs) [2,3], which poses significant challenges for data analysis. Data missingness may be attributed to a number of factors. For example, NAs may be due to compounds being truly absent in some of the biological samples, and some compounds may have concentrations below the limit of detection (LOD) of the instrument. In addition, NAs may also be caused by technical reasons such as ion suppression [4], spectral overlap, or issues related to data preprocessing. 

In general, NAs may be classified into three groups based on the patterns of data missingness, i.e., the distribution of NAs across MS-based metabolic profiles [5]. The data may be classified as missing completely at random (MCAR), missing at random (MAR), or missing not at random (MNAR). MCAR is characterized by a random distribution of NAs [6], which may also be interpreted as a lack of correlation between missingness and molecular properties. In the case of MAR, the probability of an element being missing for a particular molecule is dependent on other measured values, which may be caused by inaccurate peak detection, deconvolution of overlapping signals [7], or ion suppression. For MNAR, the occurrence of NAs is disproportionately distributed in specific molecules rather than uniformly distributed across all metabolites. For example, MNAR may be caused by compounds with concentrations below the LOD of an instrument.

In metabolomics, NAs pose a challenging issue, as a complete dataset is typically necessary for further statistical analysis. Therefore, a data preprocessing step which involves NA imputation is needed prior to data analysis. In recent decades, a number of imputation methods have been proposed, and these methods can generally be categorized into three groups. The first group imputes NAs with fixed values, such as zero, mean, median [8], minimum, or half minimum of non-missing elements in each variable. The fixed-value imputation method is technically straightforward, but it does not take into consideration that metabolites work collectively to confer a metabolic function, and thus these imputation methods may lead to estimations of NAs that deviate significantly from the ground truth, leading to bias in the subsequent analysis. 

The second class of methods imputes NAs by using global structures of data, based on the assumption that the abundance of a certain metabolite can be estimated by the abundances of the other metabolites. Examples of the second class of imputation methods include singular value decomposition (SVD) [9], probabilistic principal component analysis (PPCA) [10], and Bayesian PCA (BPCA) [11]. However, it is not straightforward to capture global structures if the data is corrupted by outliers. Thus, these methods may be sensitive to outliers, and may lead to a biased prediction of NAs in the presence of outliers. To overcome the outlier issue in NA imputation, Kumar and colleagues proposed an outlier-robust missing values imputation (ORI) [12] method which consists of SVD and an additional outlier replacement method. 

**Figure 1:**
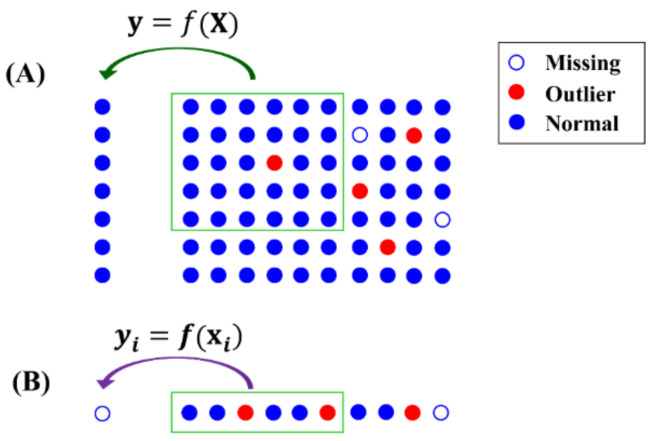
Sketch map of imputation methods based on local information. (**A**) Prediction model *f* trained using a part of data X. (**B**) NAs predicted using model f.

The third class of methods makes use of the local structures of data. As illustrated in Figure 1**,** these methods build models to predict NAs using a small portion of data ($x_{i}$) rather than the whole dataset. A portion of the data set (X) with a similar structure is selected to train the prediction model $y=f\left(X\right)$, where X might be a part of the samples and/or variables in the dataset. NAs $y_{i}$ is then imputed by the model f using the corresponding variables $x_{i}$, $y_{i}=f(x_{i})$. Random forest (RF) [13] and k-Nearest Neighbors (kNN) [14] and its variants, including NS-kNN [15] and kNN-TN [16], are methods belonging to this third class [17].

Since only a small part of data was selected to build and train the prediction model, outliers have less chance of being included in the model training. Therefore, the local structure is more robust to outliers than the global structure, in predicting NAs. However, it is not necessarily true that use of the local structure is better than use of the global structure for NA imputation. For example, if outliers are present in $x_{i}$ used for $y_{i}$ prediction (as shown in Figure 1B), it will result in a bias value $y_{i}$ even with a good prediction model. In addition, local structure is generally more heterogeneous and diverse than global structure. Sometimes, the local structure is incomplete for NA prediction, e.g., when *k* is too small in the kNN method. Taken together, NA imputation can benefit from both the local and global structures of data.

Herein, we propose a non-negative matrix factorization (NMF) [18] based approach for NA imputation. NMF can effectively capture the global structural information of the dataset by summation of the local data structures so as to preserve both global and local representation. The nonnegative constraints imposed on factor matrices in NMF make the matrix factorization self-adaptive to the local structure. In the present study, NMF imputation was carried out to generate an integrated reconstruction matrix, using weighted average over a series of reconstruction matrices, so as to minimize the impact of subjective selection on factorization based on matrix rank. Finally, the imputation capabilities and performance of the NMF-based approach were compared with three published methods (including RF, kNN, and ORI) using the corresponding metrics: NRMSE, reconstruction error of correlation coefficient network (CCN) [19], and mean score of ranking (MSR), which respectively measure the total imputation accuracy, the data structure preservation, and the performance ranking of different imputation methods. The present NMF-based approach was found capable of capturing both local and global structural characteristics of the dataset so as to be adaptive in handling different patterns of missingness, and insensitive to perturbation due to outliers. 

## 2. Results and Discussion

### 2.1. Comparison of Imputation Methods by NRMSE

Firstly, the methods were studied to quantitatively assess their imputation performance on datasets with three different patterns of NAs. The simulated datasets containing specified percentages of NAs were generated 50 times for three patterns of missingness, MAR/MCAR, MNAR, and mixed missingness (MM), which combined both types of missingness. Before imputation, data preprocessing was carried out in accordance with common practice in the metabolomics field. Those metabolites containing missing values above 30% were removed to ensure imputation was carried out on a reasonable scale [20], which is also known as the “30% deletion” rule.

**Figure 2:**
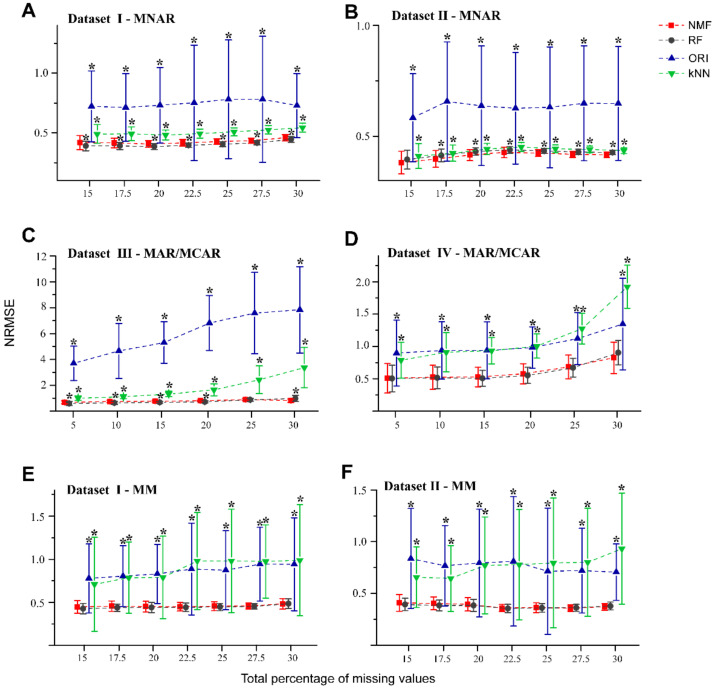
NRMSE curves obtained from the NMF, RF, ORI, and kNN methods applied on MNAR and MM patterns and MAR/MCAR patterns with different missing percentages. Fifty missingness datasets were generated randomly from Dataset I (**A**,**E**), Dataset II (**B**,**F**), Dataset III (**C**), and Dataset IV (**D**). Error bars represent the standard deviation with * denoting *p* < 0.05 (*t*-test with BH adjusted) relative to the NMF-based method.

The NRMSEs were calculated for each imputation method with increasing percentage of NAs (Figure 2). The NRMSE characterizes the total imputation accuracy between the estimated values and the observed values for each molecular feature that contains NAs. Different imputation methods produced different imputation results, depending on the traits of the dataset, missing values generation, and randomness, so that it is not straightforward to identify the perfect method for each dataset. In the MNAR cases of Figure 2A,B, NMF showed comparative performance to RF, as they both showed lower NRMSEs relative to kNN and ORI at different missing values percentages. The NRMSE for kNN was slightly higher than NMF and RF, although kNN was found to be better than ORI. Perhaps unsurprisingly, ORI was carried out using matrix factorization without the nonnegative constraint. The negative values from the matrix reconstruction probably led to high NRMSEs for ORI. For the same reason, for MAR/MCAR patterns from Dataset III (Figure 2C), the NRMSEs of ORI were found to be the highest, as compared to the other three methods at different levels of missingness.

For the cases of MAR/MCAR, kNN was found to perform better than ORI for Dataset III, but not Dataset IV (Figure 2D). This could be explained by the fact that Dataset IV was obtained from a controlled bacteria experiment which yielded lower heterogeneity and typically lower dynamic range in the data. Therefore, the impact of negative values on the NRMSE for ORI could be less than that of the increased k-neighbor variations using kNN. In the MM pattern (Figure 2E,F), the performances of NMF and RF were comparable and consistently better than kNN and ORI.

**Figure 3:**
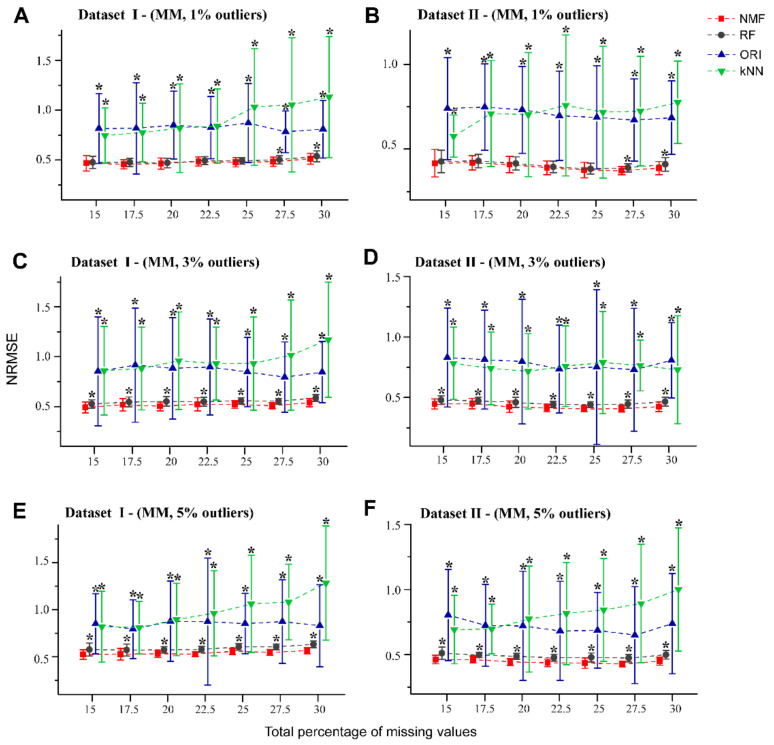
NRMSE curves for NMF, RF, kNN, and ORI apply to the MM type of missing values with 1%, 3%, and 5% outliers. Fifty missingness datasets were generated randomly from the Dataset I (**A**,**C**,**E**), and Dataset II (**B**,**D**,**F**). Error bars represent the standard deviation with * denoting *p* < 0.05 (*t*-test with BH adjusted) relative to NMF.

To assess the robustness of NMF in the presence of outliers, we used datasets I and II with different percentage of simulated NAs (in the form of MM pattern) and artificial outliers, which may be close to experimental reality. Figure 3A,B show that for both datasets containing 1% outliers, NMF performed better than RF by a narrow margin, particularly at the higher missing rates (27.5% and 30%). For datasets which contained 3% and 5% outliers (Figure 3C–F, Supplementary Figure S2A), NMF provided relatively smaller NRMSEs than RF. Consistent with the results in Figure 2, both NMF and RF showed better performance than the kNN and ORI methods in the presence of outliers. 

### 2.2. Comparison of Imputation Methods by CCN

The initial correlation coefficient networks were built from real metabolomic datasets without NAs, which were regarded as describing the original relationships among metabolites in the data. Threshold restrictions were applied to the correlation coefficients and their associated *p*-values to identify spurious correlations between metabolites. The non-significant edges were removed from CCN to ensure the sparsity of the network [21]. As a result, the sparsity of the original networks of datasets are as follows: 0.182 (Dataset I), 0.175 (Dataset II), 0.153 (Dataset III), and 0.127 (Dataset IV). For each dataset, different percentages of missing values were generated, from 5% to 30%, in steps of 5%, and included both the absence and presence of different percentages of outliers (1%, 3%, and 5%). By performing NA imputation, predicted correlation coefficient networks were constructed to observe the recovery of relationships. Precision and recall of predicted CCN were used to characterize the performance of different imputation methods. Precision indicates the ratio of true positive edges matched to the original CCN to all of the predicted edges. Recall indicates the ratio of true positive edges to all of the edges in the original CCN. In the general case, precision and recall are mutual constraints, so that the F1 score, the harmonic mean of precision and recall, is used to evaluate the integrated performance of a model. 

**Figure 4:**
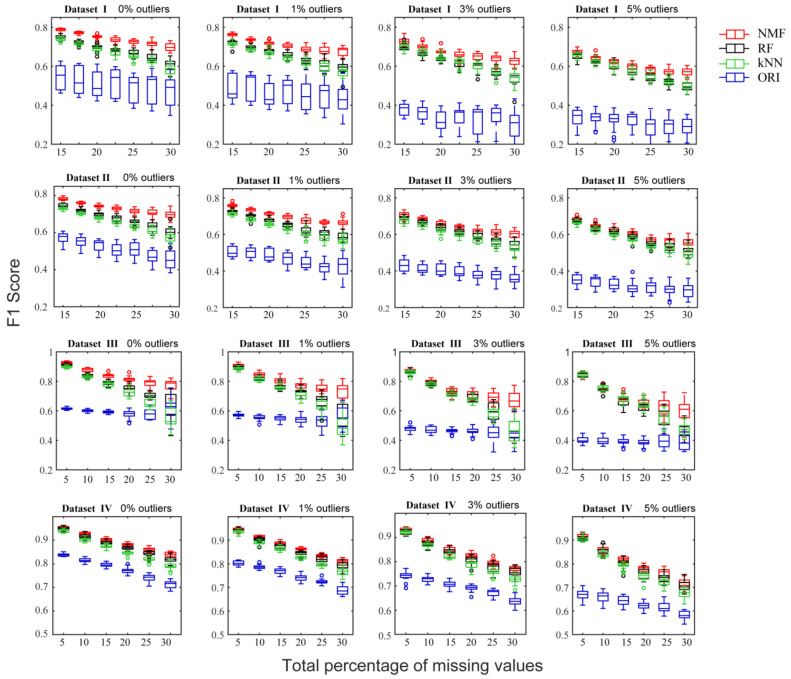
The F1 scores of predicted CCN given different rates of missing values (horizontal axis) for the NMF, RF, kNN, and ORI methods, using four metabolomics datasets.

The F1 score of predicted CCN from the datasets given different NA percentages and the different imputation methods is shown in Figure 4 and Supplementary Figure S2B. In all cases, NMF consistently produced the highest F1 score while ORI showed the lowest F1 score. In the case of 1%, 3%, and 5% outliers, the ORI algorithm showed a gentler decrease in the F1 score with increasing missing percentages, but was still the lowest relative to the other methods. Previously, the ORI method with additional outlier detection and exclusion steps was expected to be robust to outlier interference, but its drawback of high imputation errors seems to fail in preserving the original data structure.

The performance gap between NMF and RF and kNN was found to be smaller for Dataset IV, which may be explained by the dataset itself [15]. Datasets I~III were obtained from human studies and contain inherent biological variability in human samples due to various genetic and environmental factors that could lead to high data heterogeneity. On the other hand, Dataset IV was obtained from a non-human study and is expected to contain less data variation because it was performed under controlled and reproducible experimental conditions. These differences in data structure and characteristics could have unexpected impacts on the performance of imputation methods because of the inconsistency between real datasets and the assumptions made regarding data distributions underlying the algorithms. However, even for the bacterial dataset (Dataset IV), NMF still outperformed kNN and produced results comparable to, if not better than the RF method whether outliers were present or not.

### 2.3. Comparison of Imputation Methods by MSR

From Figure 2 and Figure 3, we have provided evidences showing that the NMF and RF methods were significantly better than the kNN and ORI methods from the perspective of NRMSE. Next, we carried out a further study to compare the performance of the NMF and RF methods. NRMSE measures the imputation accuracy with respect to the values of missing elements, whereas a large deviation might exist for low abundance metabolites. CCN measures the differences in associations of pairwise variables before and after imputation, whereas the existence of outliers can interfere with the measurement of the correlation coefficient in CCN. These two metrics are parametric measurements which might cause ambiguous results, especially for those imputation methods that contain considerable standard deviations. Thus, we proposed a non-parametric metric, MSR, to provide an additional evaluation of the performance of the imputation methods.

**Figure 5:**
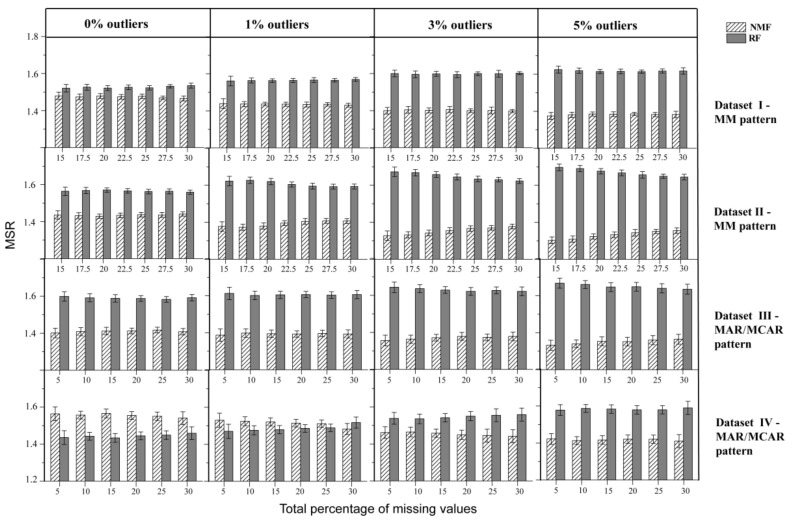
MSR for comparing the performance of NMF and RF on four datasets in the absence and presence of outliers.

In order to compare performance between NMF and RF, the MSR metric was calculated as follows. For each NA, the absolute error of the imputed values to the ground truth was calculated such that the method with the smaller absolute error was recorded as 1, or otherwise recorded as 2. MSR measured the average performance across all NAs such that the method with better performance would have an MSR value approaching 1. For Datasets I~III, NMF consistently performed better than RF, with a gradually expanding performance gap with increased levels of outliers (Figure 5). In Dataset IV, while NMF had higher MSR than RF in the absence of outliers, the situation reversed with a lower MSR for NMF with the addition of outliers. The findings suggested that the RF method may have benefited from Dataset IV, with less variation, collected under a controlled environment, but is more susceptible to perturbations by outliers. Taken together, the results of this MSR comparison indicate an advantage of NMF over RF and better robustness of NMF with respect to perturbations from outliers. This finding was further confirmed by MSR comparison for four imputation methods (Supplementary Table S1).

## 3. Datasets

### 3.1. Real-World MS-Based Metabolomics Datasets

Four experimental MS-based metabolomics datasets were used to evaluate the performance of different imputation methods in the present study. 

Dataset I and Dataset II were comprised of liquid chromatography/mass spectrometry (LC–MS) based metabolic profiles of plasma and cerebrospinal fluid, respectively, from a non-targeted metabolomics study on Alzheimer’s disease (AD). Dataset I consisted of 45 samples with 557 annotated metabolites, and Dataset II consisted of 45 samples with 476 annotated metabolites. Details regarding the experimental acquisition and clinical samples are described by Trushina et al. [22], where the data can be found under Project ID PR000045 on the Metabolomics Workbench (http://www.metabolomicsworkbench.org, accessed on 26 March 2020).

Dataset III consists of gas chromatography/mass spectrometry (GC–MS) based metabolic profiles of 56 plasma samples from a non-targeted metabolomics study of Type 2 diabetes. There are 106 metabolites identified across 56 samples. Details of the experiments are described in Fiehn et al. [23] where the data can be found under Study ID ST000383 on the Metabolomics Workbench.

Dataset IV was a GC–MS-based dataset from a study of metabolic functions of the YjgF/YER057c/UK114 (Rid) protein family of bacteria [24]. There are 249 metabolites detected in 29 sample. Data from the study were downloaded from the Metabolomics Workbench (Study ID ST000118).

Considering that datasets with large sample sizes (*n* > 100) are common in untargeted metabolomics, we further tested the imputation methods on another LC–MS/MS dataset for colorectal cancer (CRC). There are 113 metabolites identified from 234 blood samples (66 colorectal cancer patients, 76 colonic polyps patients, and 92 healthy volunteers). Data were downloaded from the Metabolomics Workbench (Project ID: PR000226).

### 3.2. Overview of Datasets

The percentages of NAs in the four published datasets, I-IV, were found to be 11.7%, 11.6%, 2%, and 0%, respectively. The distribution of missing values in Datasets I~III are shown in the first column of Supplementary Figure S1. The logarithm transformation was performed on all metabolites in all samples, and then the original data were divided evenly into 20 intervals according to the ascending log_10_(abundance). The percentage missing in each interval was calculated accordingly. For Dataset I, the highest NA percentage was observed in the second interval. On the other hand, the third interval was found to have the highest NA percentage for Dataset II. For Dataset III, the NAs are distributed in dispersed intervals, indicating a feature of MCAR. The distributions of NAs in each interval were inhomogeneous among different datasets, which may depend on multiple factors, such as peak abundance, *m*/*z* values, instrumental conditions, and preprocessing methods. 

### 3.3. Missing Values Simulation

In order to provide insight into the ground truth of NA values in a dataset and to quantify the performance of different imputation methods, observed values were randomly removed from the dataset to simulate artificial NAs. An adaptive method was proposed to simulate original NA distribution across a dataset to simulate MAR/MCAR, MNAR values, and mixed missingness (MM), which combined both types of missingness. Randomly chosen metabolites from the first two LC–MS datasets were used to generate the MNAR and MM patterns with missing percentages that ranged from 15% to 30% in steps of 2.5%; metabolites from the last two GC–MS datasets were used to generate the MAR/MCAR pattern with missing percentages that ranged from 5% to 30% in steps of 5%. 

For the MAR/MCAR simulation, metabolites present in the original dataset were randomly chosen and replaced with NAs to generate artificial missing MAR/MCAR values.

For MNAR, NAs were simulated by applying an artificial LOD to the dataset. In this case, the total missing percentage was set to x% which includes r% of original missing values and $\left(x-r\right)$% of artificial missing values. The LOD was predetermined by signal-to-noise-ratio (SNR) so that original NAs in those intervals which were lower than LOD were considered to be MNAR while the original NAs that occurred in intervals above the LOD were attributed to MAR/MCAR. The artificial NAs were simulated proportionally to the missingness of those intervals below the LOD. This approach produced similar missing trends to those of the original dataset for compounds below the LOD and kept the NA percentages of intervals above the LOD unchanged.

For MM simulation, with a predefined total NA percentage x% and given original missing percentage r%, $20\%\times \left(x-r\right)\%$ was allocated for MAR/MCAR on intervals above the LOD and the rest, $80\%\times \left(x-r\right)\%,$ was for MNAR in intervals below the LOD. Similar to MNAR, the distribution of missing percentage for either MAR/MCAR or MNAR is proportional to the NA percentage of each interval in the original data.

The second and third columns of Figure S1 show the distribution of NAs in the simulation dataset. Simulated MM patterns in datasets I and II are observed to have trends quite similar to the original data, and MNAR patterns had similar distribution of NAs below LOD. The MAR/MCAR in Dataset III and Dataset IV were characterized by a random distribution of NAs. These simulated results indicated the appropriateness of the proposed NA simulation methods.

For datasets III and IV, which were acquired using a GC–MS, we performed MAR/MCAR simulation for NAs, but not MNAR and MM. We evaluated the distributions of NAs in these real metabolomics datasets (Supplementary Figure S1), and found that NAs in GC–MS profiling data (Dataset III) are in accordance with the features of MCAR/MAR. Besides, Wei RM et al. have reported that MCAR/MAR widely occurred in GC–MS dataset [17]. Therefore, in the present study, we have only included the effect of imputation methods on the MAR/MCAR missingness present in the GC–MS datasets (Datasets III and IV).

### 3.4. Outlier Simulation

To assess the robustness of NMF-based imputation in the presence of outliers, we also randomly replaced a small part of metabolites as artificial outliers in the datasets. Three percentage levels, 1%, 3%, and 5%, of the real values in the datasets were substituted by random values from a normal distribution of $N\left(\mu_{i},\pm \;5\sigma_{i}\right)$ where $\mu_{i}$ and $\sigma_{i}$ denote the mean and the standard deviation of the i-th metabolite.

## 4. Methods

### 4.1. Non-Negative Matrix Factorization

NMF [18] is an algorithm for dimension reduction and feature extraction of high-dimensional data. It has been widely used to deal with multivariate data in various fields such as DNA gene expression analysis [25], multimedia data analysis [26], and text mining [27]. The nonnegative constraints imposed on factor matrices in the decomposition are useful in seeking more interpretable representations of data relative to the classic SVD approach. 

Let $X={\left(x_{ij}\right)}_{I\times J}$ be a non-negative abundance/concentration matrix with I metabolites and J samples. NMF aims to decompose X into the product of two non-negative matrices $B={\left(b_{ir}\right)}_{I\times K}$ and $C={\left(c_{rj}\right)}_{K\times J}$ with $K\;\left(K\geq 1\right)$ components (or basis) as follows,

(1) $$X\approx \hat{X}=B\times C$$

where $\hat{X}$ is an approximation of X. B and C are the basis and weight matrices, respectively. The NMF algorithm [18] achieves a matrix factorization by minimizing the following loss function,

(2) $$L=\|X-B\times C{\|}^{2}=\underset{ij}{\sum }{\left(x_{ij}-\overset{K}{\underset{k=1}{\sum }}b_{ik}c_{kj}\right)}^{2},\;s.t.\;B\geq 0,C\geq 0$$

when NAs are present in X, the observed value of X is partitioned into the observation set Ω

$$\Omega =\left\{\left(i,j\right)|\;x_{ij}\;is\;observed\;value\right\}$$

$\left|\Omega \right|$ is the elemental number of Ω. The observed loss function of NMF can then be written as:

(3) $$L=\|X-\hat{X}{\|}_{\left(i,j\right)\in \Omega }^{2}=\underset{\left(i,j\right)\in \Omega }{\sum }{\left(x_{ij}-\overset{K}{\underset{k=1}{\sum }}b_{ik}c_{kj}\right)}^{2},\;s.t.\;B\geq 0,C\geq 0$$

### 4.2. Manipulation of NMF-Based Imputation

Here, we proposed a new imputation algorithm based on NMF as follows:

Step 1: Initialization. Set parameter *N*. Initialize all NAs in X using a mean imputation approach, then perform logarithm transformation on X to each element, $X=\log_{10}\left(X\right)$.

Step 2: NMF factorization. For $K=k_{1}$ to $k_{N}$, calculate the reconstruction of ${\hat{X}}^{\left(K\right)}$ using the NMF algorithm with loss function Equation (3), where K is an integer, $k_{1}=max\left\{|rank\left(X\right)-\frac{N}{2}|,1\right\}$, $k_{N}=min\left\{|rank\left(X\right)+\frac{N}{2}|,J,I\right\}$, and $rank\left(X\right)$ is the rank of data matrix X. A set of reconstructed matrices $\left\{{\hat{X}}^{\left(K\right)}|K=k_{1},k_{1}+1,\cdots ,\;k_{N}\right\}$ is obtained.

Step 3: Weighted reconstruction. Calculate the reconstruction error for each K as follows,

$$d_{K}=\frac{\sum_{\left(i,j\right)\in \Omega }\left|{\hat{x}}_{ij}^{\left(K\right)}-x_{ij}\right|}{\left|\Omega \right|}$$

Then calculate the weighted reconstruction of data matrix as follows,

$$\hat{X}=\frac{\sum_{K}e^{-d_{K}}{\hat{X}}^{\left(K\right)}}{\sum_{k}e^{-d_{K}}}$$

Step 4: Imputation. Impute the NAs as follows,

$$\tilde{X}=\left({\tilde{x}}_{ij}\right)=\left\{\begin{array}{c} x_{ij\;}\left(i,j\right)\in \Omega \\ {\hat{x}}_{ij}\;\left(i,j\right)\notin \Omega \end{array}\right.$$

The above algorithm is called NMF-based imputation. It achieves an accuracy result by weighted averaging across multiple NMF models to fuse different-scale data structures in the matrix X.

Parameter N in the algorithm is the number of NMF models whose components number are different from each other, i.e., $K=k_{1},k_{1}+1,\cdots ,\;k_{N}$. Theoretically, K can be chosen between 1 and $min\left\{I,J\right\}$, but large K or large N will make the algorithm more computationally complex and more time-consuming. As a compromise on computational complexity and reconstruction errors, moderation K is selected close to the rank of data matrix, and N is set by an empirical value of 20.

### 4.3. Other Imputation Methods

The developed this NMF-based method was compared with three other imputation methods, including RF [13], kNN [14], and ORI [12] on imputation performance in the absence or presence of artificial outliers. RF [28] is a non-parametric imputation method which adopts a random forest algorithm to predict NAs of target variables based on observed values of other variables. The R package *missForest* [29] was used for this method. On the other hand, kNN imputes NAs of one target sample by averaging those non-missing elements from its k-most biologically similar samples identified based on a defined distance metric. The R package *impute* [30] was used for kNN imputation here. ORI is essentially a type of matrix factorization method which converts outliers to be NAs using outlier detection beforehand, and then estimates the NAs by minimizing the two-way empirical mean absolute error loss function [12]. 

Prior parameter optimization was carried out for kNN, RF, and ORI to reach optimal performance with the purpose of avoiding potentially biased comparisons. The optimal number of neighbors for kNN was tested and chosen to be equal to 10. On the other hand, the RF method was carried out using a nonparametric imputation tool–*missForest*. The default values of the *missForest* function were applied, with the maximum iteration set to 10 [13].

### 4.4. Evaluation Metrics

NRMSE. The imputation performance was evaluated by the normalized root mean square error (NRMSE) between the original known values and imputed ones,

$$\mathrm{NRMSE}=\frac{1}{\sqrt{\left|\Theta_{s}\right|}}\sqrt{\sum_{\left(i,j\right)\in \Theta_{s}}{\left(\frac{x_{ij}-{\tilde{x}}_{ij}}{x_{ij}}\right)}^{2}}$$

where $\Theta_{s}$ denote the simulated missing set and $\left|\Theta_{s}\right|$ is the elemental number of $\Theta_{s}$.

Network topology. Correlation coefficient network is a kind of network analysis that measures the correlation coefficient between two variables. One node in CCN represents an identified metabolite and one edge represents the association between two metabolites. Here, CCN is applied to characterize the latent structure of data and retrieve the associations between metabolites after NAs imputation. To make the datasets satisfy the completeness requirement of CCN construction, the columns with NAs were removed prior to the calculation of the precision matrix.

Different percentages of artificial NAs were generated from 5% to 30% in steps of 5%, and containing 1%, 3%, or 5% artificial outliers. By estimating NAs with NMF and other methods, CCN were built based on these imputed datasets. The precision, recall, and F1 score of predicted networks are defined as follows to evaluate the performance of imputation,

$$\mathrm{Precision}=\frac{TP}{TP+FP}$$

$$\mathrm{Recall}=\frac{TP}{TP+FN}$$

$$F1\mathrm{score}=2\times \frac{\mathrm{precision}\times \mathrm{recall}}{\mathrm{precision}+\mathrm{recall}}$$

where true positives (TP) represent the true predicted edges with respect to the reconstruction of the network without NAs, whereas false positives (FP) represent the false edges predicted after the imputation, and false negatives (FN) represent the predicted absent edges, but present in actual result in the CCN. The F1 score is a compromise between precision and recall. 

MSR. Another non-parametric metric, the mean score of ranking (MSR), was also used for the evaluation of the developed method. MSR is the absolute error of estimated NAs to the true values calculated and ranked across different imputation methods. For a given imputation method p, the ${\mathrm{MSR}}^{\left(p\right)}$ is calculated by averaging the rankings of different methods for each missing element as follows,

$${\mathrm{MSR}}^{\left(p\right)}=\frac{1}{\left|\Theta_{s}\right|}\sum_{\left(i,j\right)\in \Theta_{s}}r_{ij}^{\left(p\right)}$$

where $r_{ij}^{\left(p\right)}$ is the order of the absolute deviance between the estimated value ${\tilde{x}}_{ij}^{\left(p\right)}$ and true value $x_{ij}$, *i.e.*, $\left|x_{ij}-{\tilde{x}}_{ij}^{\left(p\right)}\right|,$ across all candidate methods p=1,2,⋯,P. This metric provides a robust and unbiased comparison which is independent of the estimated value itself.

## 5. Conclusions

We proposed a novel NMF-based scheme for NA imputation of MS-based metabolic profiles. NRMSE and F1 score for CCN and MSR were used to evaluate the performance of NMF from the perspectives of numerical accuracy of imputation, retrieval of data structures, and ordering of imputation superiority. Analysis of the simulated data in absence of outliers showed that NMF has accuracy comparable in NRMSE with the RF method, and the developed method performed better than the ORI and kNN methods. A slight advantage of NMF over RF was shown for the cases with outliers at higher percentages (3% and 5%). The results indicated the NMF method is more robust to the perturbations caused by outliers. Additionally, NMF showed the highest F1 scores for CCN at different levels of missingness in the absence or presence of outliers. In addition, the developed NMF method also produced better non-parametric MSR results than the RF method.

In summary, the performance of imputation may be affected by several factors such as patterns of missingness, specified missing percentages, numbers of outliers, and heterogeneous features of the dataset itself. However, the current results highlighted that the NMF-based method produced the overall best performance in most of the tested cases, evaluated using the three metrics of NRMSE and F1 score of CCN and MSR. In addition, NMF is robust without requiring prior knowledge on the patterns of missing data and preprocessing steps for outlier detection and exclusion. Therefore, this proposed method may be a useful missing data imputation in metabolomics. 

## Data Availability

R script for the proposed NMF based imputation method is available at https://github.com/freeoliver-jing/NMF (accessed on 10 September 2021).

## Supplementary Materials

Figure S1: The distribution of NAs in real datasets. Figure S2: Imputation results for the metabolomics dataset of colorectal cancer. Figure S3: Changed metabolites before and after NAs imputation. Table S1: Performance of four different methods on Dataset I-IV and CRC dataset.

## Sample Availability

Samples of the compounds are available from the authors.
