# Supplementary material for: NMF-Based Approach for Missing Values Imputation of Mass Spectrometry Metabolomics Data
Source: Molecules. 2021 Sep 24;26(19):5787. doi: 10.3390/molecules26195787 (PMC8510447; doi:10.3390/molecules26195787)
Supplement: Supplementary file 1 [file molecules-26-05787-s001.zip › molecules-1337627-supplementary.pdf]

*Supplementary Materials*

# NMF-based Approach for Missing Values Imputation of Mass Spectrometry Metabolomics Data

**Jingjing Xu [1], Yuanshan Wang [1], Xiangnan Xu [2], Kian-Kai Cheng [3], Daniel Raftery [4], Jiyang Dong [1,5*]**

[1] Department of Electronic Science, Xiamen University, Xiamen 361005, China; jingjing@xmu.edu.cn (J. X.); 574428960@qq.com (Y. W.); jydong@xmu.edu.cn (J. D.)
[2] School of Mathematics and Statistics, The University of Sydney, NSW 2006, Australia; xiangnan.xu@sydney.edu.au (X. X.)
[3] Innovation Centre in Agritechnology, Universiti Teknologi Malaysia, Johor, Muar 84600, Malaysia; chengkiankai@cheme.utm.my (K. C.)
[4] Northwest Metabolomics Research Center, Department of Anesthesiology and Pain Medicine, University of Washington, Seattle, WA 98109, USA; draftery@uw.edu (D. R.)
[5] National Institute for Data Science in Health and Medicine, Xiamen University, Xiamen 361005, China
* Correspondence: jydong@xmu.edu.cn; Tel.: 86-592-2183301

**Figure S1. The distribution of NAs in real datasets. (pp. S2)**

**Figure S2. Imputation results for the metabolomics dataset of colorectal cancer. (pp.S3)**

**Figure S3. Changed metabolites before and after NAs imputation. (pp.S4)**

**Table S1. Performance of four different methods on Dataset I-IV and CRC dataset. (pp.S5-S9)**

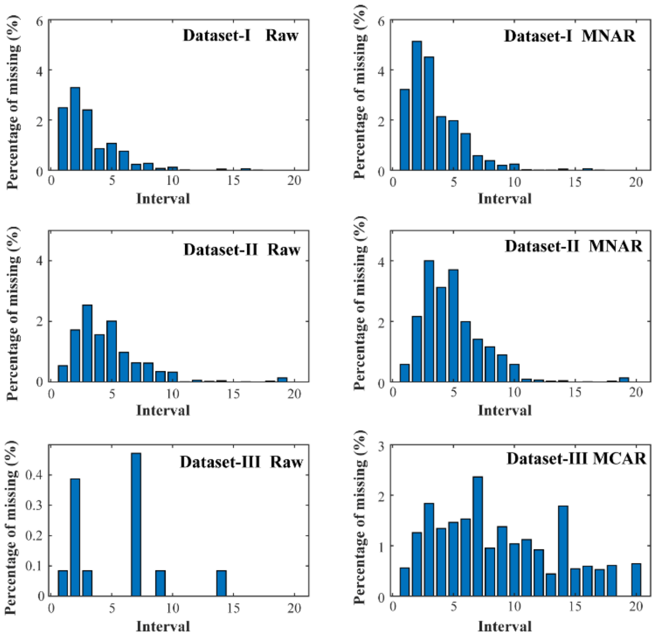


**Figure S1. The distribution of NAs in real datasets.** Histograms in the first column shows the ratio of raw missingness in each interval according to the ascending lg(abundance) of metabolites. The second and third column represent the simulation missing patterns of each dataset, and total missing percentage is predetermined to 20%.

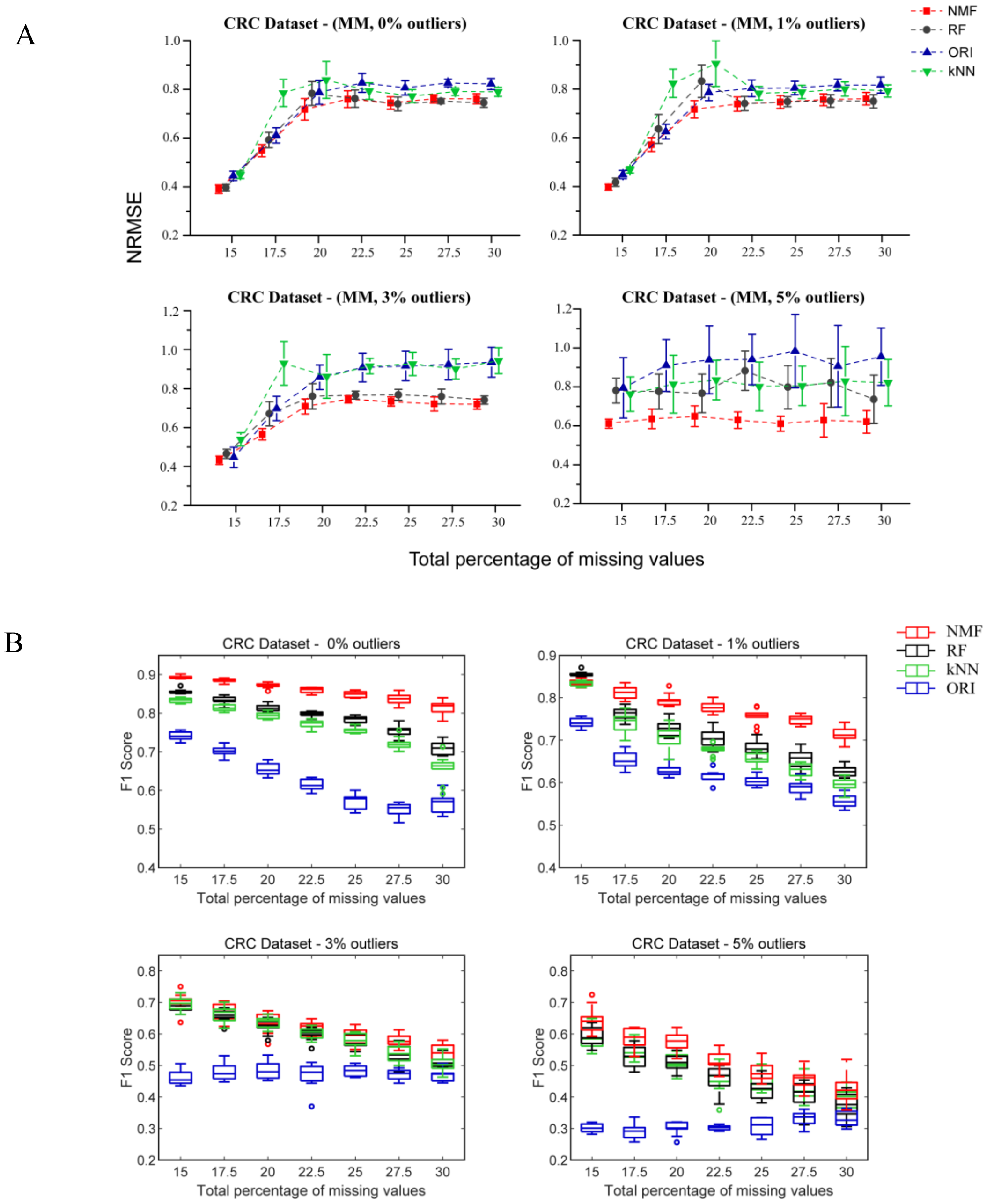


**Figure S2. Imputation results for the metabolomics dataset of colorectal cancer.** NRMSE curves (A) and F1 Score (B) for NMF, RF, kNN and ORI applies to the MM types of missing values with 0%, 1%, 3% and 5% outliers. Fifty missingness datasets were generated randomly from the metabolomics dataset of colorectal cancer (CRC).

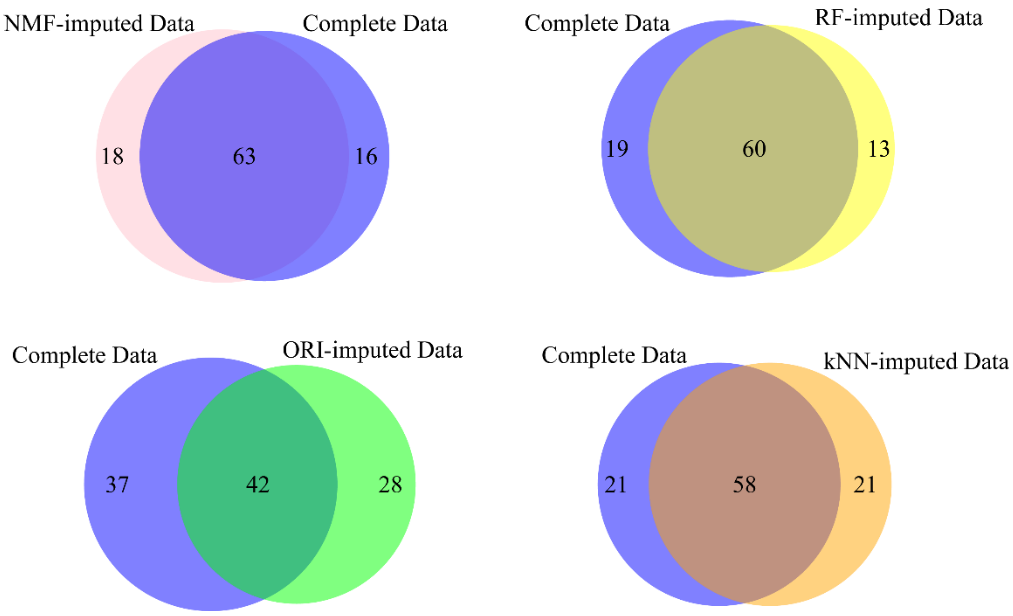


**Figure S3. Changed metabolites before and after NAs imputation.** Number of metabolites identified to be differential expression (DE) among two groups of Dataset I using supervised PLS-DA**.** The DE metabolites from complete data were compared to NMF-, RF-, ORI- and kNN-imputed Data.

**Table S1.** MSR for comparing the performance of four methods on Dataset **I-IV** and CRC dataset.

| Percentage of Outliers \ Percentage of Missing | | Dataset I | | | | | | |
|---|---|---|---|---|---|---|---|---|
| | | **15%** | **17.5%** | **20%** | **22.5%** | **25%** | **27.5%** | **30%** |
| **0%** | NMF | 2.452±0.046 | 2.460±0.025 | 2.461±0.033 | 2.447±0.023 | 2.446±0.024 | 2.444±0.028 | 2.429±0.027 |
| | RF | 2.473±0.041 | 2.460±0.043 | 2.474±0.031 | 2.478±0.041 | 2.476±0.030 | 2.483±0.045 | 2.512±0.030 |
| | ORI | 2.557±0.041 | 2.555±0.028 | 2.533±0.026 | 2.541±0.029 | 2.540±0.027 | 2.532±0.032 | 2.533±0.022 |
| | kNN | 2.519±0.050 | 2.525±0.027 | 2.532±0.032 | 2.534±0.028 | 2.538±0.027 | 2.541±0.034 | 2.527±0.020 |
| **1%** | NMF | 2.354±0.050 | 2.392±0.038 | 2.395±0.050 | 2.384±0.041 | 2.374±0.048 | 2.376±0.039 | 2.380±0.036 |
| | RF | 2.528±0.047 | 2.504±0.040 | 2.521±0.024 | 2.516±0.032 | 2.509±0.028 | 2.507±0.031 | 2.506±0.029 |
| | ORI | 2.582±0.045 | 2.569±0.027 | 2.560±0.032 | 2.572±0.029 | 2.574±0.043 | 2.569±0.029 | 2.562±0.024 |
| | kNN | 2.535±0.051 | 2.535±0.032 | 2.525±0.034 | 2.529±0.025 | 2.543±0.029 | 2.549±0.022 | 2.552±0.025 |
| **3%** | NMF | 2.211±0.043 | 2.209±0.039 | 2.207±0.022 | 2.225±0.039 | 2.220±0.036 | 2.234±0.04 | 2.232±0.043 |
| | RF | 2.535±0.045 | 2.524±0.041 | 2.554±0.035 | 2.542±0.026 | 2.549±0.033 | 2.556±0.03 | 2.557±0.035 |
| | ORI | 2.638±0.031 | 2.643±0.041 | 2.622±0.028 | 2.618±0.045 | 2.624±0.035 | 2.611±0.037 | 2.615±0.043 |
| | kNN | 2.616±0.051 | 2.624±0.039 | 2.617±0.039 | 2.616±0.041 | 2.607±0.022 | 2.599±0.029 | 2.596±0.025 |
| **5%** | NMF | 2.128±0.059 | 2.107±0.035 | 2.083±0.034 | 2.099±0.027 | 2.101±0.032 | 2.114±0.032 | 2.111±0.030 |
| | RF | 2.536±0.051 | 2.532±0.03 | 2.551±0.028 | 2.552±0.035 | 2.553±0.031 | 2.560±0.027 | 2.558±0.027 |
| | ORI | 2.718±0.052 | 2.725±0.033 | 2.730±0.026 | 2.717±0.036 | 2.715±0.023 | 2.705±0.033 | 2.688±0.025 |
| | kNN | 2.618±0.047 | 2.636±0.038 | 2.636±0.034 | 2.632±0.023 | 2.632±0.030 | 2.620±0.031 | 2.642±0.023 |

| Percentage of Missing / Percentage of Outliers | | Dataset II | | | | | | |
|---|---|---|---|---|---|---|---|---|
| | | **15%** | **17.5%** | **20%** | **22.5%** | **25%** | **27.5%** | **30%** |
| **0%** | NMF | 2.413±0.021 | 2.453±0.024 | 2.435±0.032 | 2.439±0.031 | 2.428±0.019 | 2.440±0.024 | 2.422±0.037 |
| | RF | 2.489±0.041 | 2.476±0.060 | 2.485±0.035 | 2.485±0.053 | 2.487±0.044 | 2.459±0.035 | 2.457±0.053 |
| | ORI | 2.561±0.044 | 2.565±0.051 | 2.571±0.041 | 2.578±0.025 | 2.588±0.029 | 2.578±0.013 | 2.580±0.020 |
| | kNN | 2.537±0.076 | 2.506±0.030 | 2.509±0.057 | 2.498±0.031 | 2.497±0.022 | 2.523±0.021 | 2.540±0.033 |
| **1%** | NMF | 2.320±0.046 | 2.332±0.033 | 2.331±0.033 | 2.332±0.034 | 2.317±0.023 | 2.324±0.026 | 2.316±0.044 |
| | RF | 2.507±0.038 | 2.480±0.031 | 2.522±0.026 | 2.504±0.035 | 2.518±0.036 | 2.520±0.027 | 2.516±0.043 |
| | ORI | 2.620±0.032 | 2.631±0.060 | 2.604±0.026 | 2.640±0.024 | 2.623±0.031 | 2.614±0.020 | 2.530±0.020 |
| | kNN | 2.553±0.050 | 2.557±0.020 | 2.542±0.036 | 2.523±0.022 | 2.541±0.034 | 2.542±0.023 | 2.638±0.026 |
| **3%** | NMF | 2.189±0.061 | 2.207±0.049 | 2.194±0.042 | 2.199±0.046 | 2.180±0.050 | 2.166±0.05 | 2.187±0.041 |
| | RF | 2.531±0.059 | 2.492±0.037 | 2.490±0.036 | 2.515±0.035 | 2.505±0.032 | 2.523±0.031 | 2.532±0.030 |
| | ORI | 2.668±0.051 | 2.681±0.031 | 2.681±0.043 | 2.674±0.027 | 2.690±0.030 | 2.678±0.026 | 2.673±0.029 |
| | kNN | 2.612±0.042 | 2.620±0.035 | 2.635±0.023 | 2.611±0.027 | 2.625±0.035 | 2.633±0.027 | 2.608±0.021 |
| **5%** | NMF | 2.068±0.051 | 2.069±0.056 | 2.084±0.053 | 2.078±0.041 | 2.068±0.033 | 2.055±0.032 | 2.093±0.046 |
| | RF | 2.494±0.054 | 2.523±0.031 | 2.496±0.031 | 2.515±0.036 | 2.521±0.030 | 2.538±0.033 | 2.529±0.026 |
| | ORI | 2.744±0.059 | 2.717±0.066 | 2.717±0.042 | 2.715±0.030 | 2.707±0.021 | 2.719±0.038 | 2.690±0.033 |
| | kNN | 2.693±0.067 | 2.691±0.041 | 2.704±0.035 | 2.691±0.034 | 2.705±0.041 | 2.687±0.017 | 2.688±0.044 |

| Percentage of Outliers \ Percentage of Missing | | Dataset III | | | | | |
|---|---|---|---|---|---|---|---|
| | | **5%** | **10%** | **15%** | **20%** | **25%** | **30%** |
| **0%** | NMF | 2.225±0.026 | 2.269±0.026 | 2.260±0.027 | 2.263±0.033 | 2.238±0.024 | 2.194±0.036 |
| | RF | 2.483±0.057 | 2.357±0.023 | 2.379±0.032 | 2.413±0.052 | 2.440±0.071 | 2.426±0.057 |
| | ORI | 2.772±0.055 | 2.781±0.059 | 2.768±0.025 | 2.763±0.032 | 2.795±0.081 | 2.883±0.067 |
| | kNN | 2.521±0.018 | 2.593±0.031 | 2.593±0.021 | 2.561±0.016 | 2.527±0.014 | 2.497±0.026 |
| **1%** | NMF | 2.284±0.025 | 2.223±0.032 | 2.286±0.035 | 2.322±0.039 | 2.322±0.012 | 2.256±0.043 |
| | RF | 2.359±0.031 | 2.396±0.045 | 2.305±0.059 | 2.345±0.043 | 2.342±0.044 | 2.294±0.570 |
| | ORI | 2.810±0.044 | 2.781±0.059 | 2.770±0.024 | 2.779±0.021 | 2.772±0.038 | 2.924±0.038 |
| | kNN | 2.546±0.029 | 2.600±0.036 | 2.638±0.031 | 2.554±0.021 | 2.564±0.032 | 2.526±0.026 |
| **3%** | NMF | 2.099±0.035 | 2.002±0.018 | 2.033±0.024 | 2.086±0.016 | 2.180±0.027 | 2.037±0.026 |
| | RF | 2.449±0.041 | 2.486±0.036 | 2.490±0.048 | 2.461±0.038 | 2.518±0.025 | 2.536±0.021 |
| | ORI | 2.741±0.052 | 2.799±0.030 | 2.754±0.055 | 2.783±0.029 | 2.719±0.037 | 2.809±0.030 |
| | kNN | 2.713±0.027 | 2.712±0.043 | 2.732±0.035 | 2.670±0.043 | 2.583±0.040 | 2.618±0.038 |
| **5%** | NMF | 1.964±0.019 | 1.872±0.025 | 1.919±0.020 | 1.976±0.032 | 1.954±0.017 | 1.883±0.022 |
| | RF | 2.480±0.024 | 2.550±0.039 | 2.608±0.023 | 2.508±0.034 | 2.585±0.024 | 2.586±0.031 |
| | ORI | 2.832±0.032 | 2.808±0.042 | 2.755±0.032 | 2.789±0.044 | 2.749±0.033 | 2.864±0.048 |
| | kNN | 2.724±0.034 | 2.770±0.028 | 2.718±0.019 | 2.727±0.040 | 2.712±0.026 | 2.667±0.025 |

| Percentage of Outliers \ Percentage of Missing | | Dataset IV | | | | | |
| --- | --- | --- | --- | --- | --- | --- | --- |
| | | **5%** | **10%** | **15%** | **20%** | **25%** | **30%** |
| **0%** | NMF | 2.184±0.023 | 2.268±0.035 | 2.195±0.020 | 2.176±0.026 | 2.333±0.029 | 2.372±0.034 |
| | RF | 2.346±0.036 | 2.486±0.027 | 2.502±0.030 | 2.521±0.036 | 2.412±0.021 | 2.461±0.025 |
| | ORI | 2.773±0.051 | 2.677±0.038 | 2.732±0.044 | 2.694±0.052 | 2.631±0.026 | 2.680±0.048 |
| | kNN | 2.697±0.030 | 2.569±0.033 | 2.571±0.037 | 2.609±0.022 | 2.624±0.030 | 2.487±0.028 |
| **1%** | NMF | 2.238±0.014 | 2.240±0.039 | 2.274±0.017 | 2.301±0.029 | 2.417±0.025 | 2.399±0.018 |
| | RF | 2.384±0.027 | 2.539±0.026 | 2.510±0.030 | 2.543±0.018 | 2.430±0.035 | 2.409±0.037 |
| | ORI | 2.708±0.040 | 2.616±0.028 | 2.633±0.035 | 2.613±0.049 | 2.632±0.034 | 2.716±0.032 |
| | kNN | 2.670±0.038 | 2.605±0.037 | 2.583±0.026 | 2.543±0.051 | 2.520±0.021 | 2.476±0.037 |
| **3%** | NMF | 2.189±0.020 | 2.317±0.030 | 2.434±0.033 | 2.337±0.026 | 2.334±0.036 | 2.294±0.038 |
| | RF | 2.519±0.034 | 2.512±0.029 | 2.488±0.039 | 2.464±0.025 | 2.455±0.022 | 2.440±0.027 |
| | ORI | 2.665±0.042 | 2.601±0.040 | 2.560±0.027 | 2.632±0.037 | 2.674±0.031 | 2.704±0.044 |
| | kNN | 2.627±0.031 | 2.573±0.036 | 2.519±0.035 | 2.567±0.029 | 2.537±0.033 | 2.562±0.028 |
| **5%** | NMF | 2.281±0.018 | 2.355±0.028 | 2.267±0.032 | 2.243±0.040 | 2.251±0.037 | 2.214±0.035 |
| | RF | 2.465±0.025 | 2.501±0.021 | 2.552±0.028 | 2.546±0.041 | 2.514±0.032 | 2.483±0.024 |
| | ORI | 2.692±0.036 | 2.609±0.034 | 2.623±0.049 | 2.641±0.038 | 2.704±0.029 | 2.670±0.039 |
| | kNN | 2.562±0.032 | 2.534±0.026 | 2.558±0.038 | 2.569±0.051 | 2.531±0.030 | 2.634±0.027 |

| Percentage of Outliers \ Percentage of Missing | | CRC Dataset | | | | | | |
|---|---|---|---|---|---|---|---|---|
| | | **15%** | **17.5%** | **20%** | **22.5%** | **25%** | **27.5%** | **30%** |
| **0%** | NMF | 2.309±0.035 | 2.282±0.034 | 2.256±0.035 | 2.218±0.041 | 2.219±0.045 | 2.195±0.041 | 2.245±0.033 |
| | RF | 2.398±0.031 | 2.401±0.025 | 2.387±0.030 | 2.398±0.028 | 2.361±0.032 | 2.34±0.025 | 2.333±0.023 |
| | ORI | 2.711±0.018 | 2.717±0.035 | 2.737±0.041 | 2.734±0.033 | 2.719±0.020 | 2.779±0.045 | 2.745±0.038 |
| | kNN | 2.582±0.053 | 2.601±0.022 | 2.619±0.048 | 2.649±0.051 | 2.700±0.054 | 2.686±0.026 | 2.677±0.043 |
| **1%** | NMF | 1.959±0.061 | 1.961±0.067 | 1.950±0.061 | 1.964±0.058 | 1.951±0.068 | 2.008±0.033 | 1.975±0.046 |
| | RF | 2.595±0.041 | 2.585±0.038 | 2.583±0.04 | 2.571±0.049 | 2.547±0.036 | 2.520±0.047 | 2.558±0.052 |
| | ORI | 2.839±0.038 | 2.785±0.032 | 2.804±0.042 | 2.796±0.064 | 2.766±0.08 | 2.743±0.034 | 2.736±0.064 |
| | kNN | 2.606±0.083 | 2.669±0.048 | 2.662±0.064 | 2.669±0.089 | 2.736±0.047 | 2.729±0.064 | 2.732±0.085 |
| **3%** | NMF | 1.698±0.046 | 1.721±0.032 | 1.709±0.061 | 1.755±0.017 | 1.777±0.067 | 1.831±0.042 | 1.821±0.031 |
| | RF | 2.653±0.076 | 2.660±0.059 | 2.652±0.080 | 2.669±0.055 | 2.660±0.051 | 2.691±0.042 | 2.654±0.055 |
| | ORI | 2.990±0.051 | 2.940±0.059 | 2.975±0.069 | 2.899±0.05 | 2.874±0.037 | 2.786±0.025 | 2.778±0.046 |
| | kNN | 2.659±0.041 | 2.679±0.034 | 2.665±0.034 | 2.676±0.048 | 2.689±0.064 | 2.692±0.035 | 2.746±0.058 |
| **5%** | NMF | 1.587±0.032 | 1.652±0.047 | 1.636±0.054 | 1.631±0.034 | 1.743±0.043 | 1.738±0.040 | 1.760±0.022 |
| | RF | 2.628±0.054 | 2.562±0.056 | 2.592±0.069 | 2.629±0.082 | 2.613±0.051 | 2.652±0.079 | 2.645±0.074 |
| | ORI | 2.710±0.032 | 3.032±0.045 | 3.040±0.067 | 2.980±0.049 | 2.897±0.060 | 2.875±0.029 | 2.827±0.080 |
| | kNN | 3.075±0.035 | 2.756±0.051 | 2.732±0.048 | 2.761±0.045 | 2.747±0.044 | 2.736±0.037 | 2.768±0.053 |

## Difference between ORI and NMF algorithms.

There are some differences between ORI and NMF as follows,

Firstly, ORI decomposes the data matrix into two vectors (one component, $K = 1$) by means of singular value decomposition (SVD). Restricted by the dimension of matrix decomposition, ORI is apt to capture the global structure of data but failed on obtaining elaborated information. NMF is capable of both global and local representation of data by weighted average over a series of reconstruction matrix from multiple $K$.

Secondly, the nonnegative constraints in NMF does not allow negative entries in matrix factors. It is favorable to retrieve information by parts-based representation [Lee DD, Seung HS. ***Nature***, 401: 788-791].

Thirdly, the ORI algorithm adopts the mean absolution error (MAE) as the loss function, which is sensitive to outlier interference. Hence it requires an additional outlier detection prior to matrix decomposition to ensure rational NAs estimation. NMF utilizes local structure of data that is more robust to outliers than global structure so as to perform better than ORI in the presence of outliers.
